# The incremental value of liquid biopsy in the initial evaluation of patients with metastatic non-small cell lung cancer undergoing tissue-based molecular testing

**DOI:** 10.1016/j.jlb.2026.100461

**Published:** 2026-03-24

**Authors:** Benjamin A. Bleiberg, Dylan G. Scholes, Lauren Reed-Guy, Devaki Dravid, Lova L. Sun, Aditi P. Singh, Roger B. Cohen, Corey J. Langer, Melina E. Marmarelis, Charu Aggarwal

**Affiliations:** a Division of Hematology-Oncology, Department of Medicine, Perelman School of Medicine, University of Pennsylvania, Philadelphia, USA; b Abramson Cancer Center, University of Pennsylvania, Philadelphia, USA; c Penn Center for Cancer Care Innovation, Philadelphia, PA, USA

**Keywords:** Molecular testing, NSCLC, ctDNA, Targeted therapy

## Abstract

**Introduction:**

Actionable genetic alterations (AGAs) are present in many patients with non-squamous, metastatic non-small cell lung cancer (mNSCLC) and guide the selection of targeted therapies. Guidelines support concurrent tissue- and plasma-based molecular sequencing at diagnosis to optimize detection of AGAs. Some patients with mNSCLC have alterations detected in plasma-based molecular testing but not in tissue. The mechanisms underlying this discordance, and associated patient- and tumor-specific features remain uncertain.

**Methods:**

We conducted a single-center retrospective cohort study using electronic health record data. The cohort included treatment-naïve patients with newly diagnosed non-squamous mNSCLC who underwent concurrent tissue- and plasma-based molecular sequencing between 01/01/2019 and 3/31/2022.

**Results:**

358 patients underwent concurrent tissue- and plasma-based molecular sequencing, 173 (48.3%) were found to harbor AGAs. 113 (65.3%) had alterations identified on both tissue- and plasma-based testing, 42 (24.3%) in tissue-based testing alone, and 18 (10.4%) in plasma-based testing alone. Amongst these 18 patients, 9 (50%) had AGAs in plasma-based sequencing alone due to tissue samples unsuitable for comprehensive testing, 4 (22%) had incomplete tissue-based molecular sequencing panels ordered, and 5 (28%) had no AGAs detected in tissue-based sequencing despite appropriate testing on adequate samples, constituting true tissue-negative cases. The disease control rate by RECIST 1.1 criteria was 100% for the 6 patients with plasma-only AGAs treated with first-line targeted therapy.

**Conclusions:**

Concurrent tissue- and plasma-based molecular profiling enhances AGA detection in patients with non-squamous mNSCLC. Concurrent testing may compensate for inadequate solid tumor tissue samples and tumor heterogeneity to enhance the comprehensiveness of molecular sequencing.

## Highlights

• 10% (18/173) of mNSCLC patients undergoing tissue and plasma-based molecular testing had AGAs detected only by plasma NGS.
• 72% (13/18) of these cases were attributable to inadequate tissue samples or incomplete tissue-based sequencing.
• 2.8% (5/173) had AGAs identified only in plasma-based testing despite comprehensive tissue testing on an adequate biopsy.
• Disease control rate with targeted therapy was 100% (6/6) for patients with AGAs identified only in plasma-based testing.

## Abbreviations

AGA: actionable genetic alteration
mNSCLC: metastatic non-small cell lung cancer

## 1 Introduction

Actionable genetic alterations (AGAs) are present in 45-60% of patients with mNSCLC with non-squamous histology [[1], [2], [3]]. Studies have demonstrated that obtaining plasma-based molecular sequencing prior to the establishment of a tissue diagnosis can expedite the identification of AGAs [4]. The availability of sequencing results prior to first-line treatment initiation and the utilization of appropriate targeted therapy if AGAs are detected has been associated with improved overall survival and more cost-effective care [[5], [6], [7]]. National and international society guidelines support the use of concurrent tissue- and plasma-based molecular testing at the time of initial diagnosis for all patients with mNSCLC [8,9]. This approach is associated with improved detection of AGAs compared to either sequencing approach alone. In summary, concurrent tissue- and plasma-based sequencing improves and expedites detection of AGAs and allows for the rapid implementation of the most efficacious, personalized treatments for patients with mNSCLC.

A recent study by Iams et al. (2024) found that 5.3% (27/513) of patients with mNSCLC undergoing concurrent tissue- and plasma-based sequencing had AGAs detected in plasma-based testing alone [10]. This study provided additional data supporting the growing consensus that concurrent tissue- and plasma-based testing maximizes the detection of AGAs. However, the specific characteristics of patients and the features of their somatic mutational profile that may contribute to the detection of AGAs exclusively in plasma-based assays has not been previously explored.

While physicians may order molecular testing on biopsy samples with the assumption that this testing is comprehensive, there are several pitfalls of tissue-based sequencing that we wish to explore, such as the type of testing ordered and comprehensiveness and adequacy of samples to complete sequencing. Further exploration of which patients have AGAs detected exclusively in plasma-based sequencing may allow clinicians to more clearly identify the etiology of discordant tissue- and plasma-based testing results and optimize testing strategies to increase AGA detection. Our study aims to describe the incremental value of plasma-based testing in identifying AGAs for patients undergoing tissue-based testing. Additionally, we aim to describe on a case-by-case basis the specific limitations of tissue-based testing that led to discordant tissue- and plasma-based sequencing results in our cohort.

## 2 Methods

We conducted a single-center real-world retrospective cohort study using electronic health record (EHR: Epic Systems, Madison, WI) data at the University of Pennsylvania through an institutional review board-approved protocol (IRB protocol #: 843355). The cohort included treatment-naïve patients with newly diagnosed mNSCLC with non-squamous histology who underwent tissue- and plasma-based molecular sequencing at time of initial diagnosis between 01/01/2019 and 3/31/2022. Tissue-based testing was completed using in-house and proprietary next-generation DNA and RNA sequencing (Comprehensive Solid Tumor HaloPlex^HS^, version 2.0; Agilent Technology, Inc). Plasma-based molecular sequencing utilized the proprietary Guardant360 platform from Guardant Health with an established concordance of >77% with tissue-based testing [11,12]. Blood samples were collected from patients in clinic as part of routine care and mailed to an outside lab as per Guardant Health's established protocols.

For the purposes of cohort identification via electronic health record query, patients were considered to have had concurrent testing if both tissue- and plasma-based sequencing were ordered within 60 days of the initial tissue diagnosis. This duration was chosen, given the pragmatic, retrospective nature of the study with a focus on concurrence and mechanisms of discrepancy rather than turn-around-time. This more permissive definition allowed for the inclusion of patients with prolonged hospitalizations for which plasma-based testing was not always feasible or those seeking second-opinions at a quaternary care center who may have had a tissue diagnosis of NSCLC but had not undergone comprehensive tissue- or plasma-based sequencing. To be included, patients must have had ≥1 follow-up visit after initial consultation at the study center. We identified patients with discordant results in tissue- and plasma-based sequencing, with a focus on cases in which an AGA was identified in plasma- but not in tissue-based testing. We then assessed the reasons for this discordance by individual chart review. Patients with discordant tissue- and plasma-based sequencing results were categorized into 3 discrete groups: 1) “incomplete test,” wherein ≥1 component of the tissue-based panel was not completed due to insufficient tissue sample quality or quantity 2) “inadequate panel” where only a limited tissue-based panel (absence of ≥1 of FISH, DNA, or RNA testing or use of an assay that omitted ≥1 relevant AGA) was ordered and 3) “true tissue-negative” where the relevant tissue-based panels were completed on an adequate sample, but the AGA was not detected. Demographic data, Eastern Cooperative Oncology Group Performance Status (ECOG PS), tobacco exposure, and first-line therapy data was collected on this patient subgroup.

## 3 Results

**Fig. 1:**
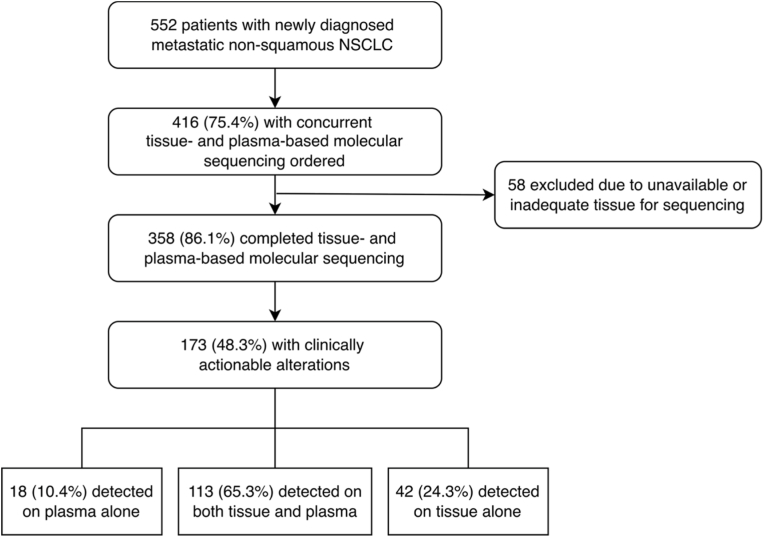
Participant flow diagram.

Among 552 patients with newly diagnosed non-squamous mNSCLC treated at our center between 1/01/2019 and 3/31/2022 (Fig. 1), 416 attempted to undergo concurrent tissue- and plasma-based molecular sequencing. Of these patients, 58 (13.9%) had unavailable or inadequate tissue to send for sequencing and were excluded from further analysis. 358 (86.1%) patients underwent tissue- and plasma-based testing, with 173 AGAs detected, representing 48.3% of individuals with concurrent testing. Of individuals with AGAs detected, 113 (65.3%) had concordant AGA detection in tissue- and plasma-based sequencing, 42 (24.3%) had AGAs detected in tissue-based testing alone, and 18 (10.4%) had AGAs detected in plasma-based testing alone. For patients with AGAs appreciated exclusively on plasma-based sequencing, time between obtaining diagnostic biopsy tissue and collection of peripheral blood for plasma-based sequencing averaged 14.6 days with a median of 13.5 days and interquartile range: 7-19 days.

Of the 18 patients with newly diagnosed non-squamous mNSCLC and AGAs found in plasma-based sequencing alone; nine (50%) had incomplete testing in which there was insufficient tissue to complete comprehensive testing. Four (22%) patients had an inadequate panel wherein essential components of comprehensive sequencing were not ordered. In some cases, even if testing was ordered, the assays performed did not include clinically relevant AGAs. Five (28%) patients had true tissue-negative results in which the AGA of interest was not detected on an appropriate solid tumor sample following comprehensive sequencing.

Demographic features of the cohort of patients with AGAs detected exclusively in plasma-based testing are presented in Table 1. The mean age for this subgroup was 65.3 years, 61% were female, 45% identified as non-White race, 75% had an ECOG PS < 2, and 66.7% were current or former smokers. Stage at time of molecular testing was IIIB in 11% (2/18) of cases, IVA in 33% (6/18), and IVB in 56% (10/18). Alterations identified included mutations in *EGFR* (2 exon 20, 2 exon 19, 1 exon 18), 4 *ERBB2* alterations, 3 *KRAS* G12C mutations, 2 *ALK* fusions, 2 *MET* exon 14 skipping alterations, 1 *RET* fusion, and 1 *BRAF* V600E mutation. Variant allele fractions for these alterations in the cohort of patients with AGAs detected exclusively in plasma-based testing are presented in Table 1 and ranged from 0.03% to 30.8% and were between 0.03% and 2.9% in the true tissue-negative subgroup. 6/18 (33%) received guideline concordant first-line targeted therapy following detection of an alteration only appreciated in plasma-based molecular sequencing. Of these patients, best overall response by Response Evaluation Criteria in Solid Tumors (RECIST) 1.1 was a partial response in 4/6 and stable disease in 2/6 cases, with an overall disease control rate of 100%. Of the remaining 12/18 patients, 9 did not have approved first-line targetable therapies available at time of diagnosis and 3 transitioned to hospice or died prior to treatment initiation. Of the 9 patients without approved targeted therapies 1L therapy consisted of combined chemotherapy and immunotherapy (n = 6), 2 received chemotherapy alone, and 1 received immunotherapy alone.

**Table 1:** Characteristics of patients with plasma-based molecular sequencing exclusive alterations.

Age	Gender	Race	ECOG PS	Stage	Smoking History	Biopsy Site	Alteration Type	Gene (Alteration)	Pathogenic Variant Allele Fraction	Reason for Discrepancy	Therapy
47	M	Black	0	IVB	Never	Bone	Insertion	*ERBB2* (Exon 20 ins)	0.8%	True Tissue-Negative	Chemo + IO
74	F	White	1	IVB	Current	Lung	Fusion	*ALK* (STRN Fusion)	0.1%	True Tissue-Negative	Hospice
68	F	Black	1	IVB	Former	Lung	Insertion	*EGFR* (Exon 20ins)	2.9%	True Tissue-Negative	Chemo
59	F	White	0	IVA	Former	Lung	Skipping	*MET* (Exon 14)	0.03%	True Tissue-Negative	Targeted
66	F	Unknown	0	IVB	Never	Lung	Insertion	*EGFR* (Exon 20ins)	0.6%	True Tissue-Negative	Chemo + IO
58	M	Black	3	IVB	Current	Lung	Deletion	*EGFR* (Exon 19del)	3.5%	Incomplete Testing	Targeted
88	M	White	2	IIIB	Former	LN	Skipping	*MET* (Exon 14)	22%	Incomplete Testing	Chemo
68	M	White	1	IVA	Never	LN	Fusion	*ALK* (EML4 Fusion)	6.4%	Incomplete Testing	Targeted
62	M	Asian	0	IVA	Never	Lung	Deletion	*EGFR* (Exon 19del)	0.07%	Incomplete Testing	Targeted
60	F	Unknown	1	IIIB	Never	LN	Fusion	*RET* (KIF5B Fusion)	3.6%	Incomplete Testing	Targeted
78	M	White	0	IVB	Former	Lung	Insertion	*ERBB2* (Exon 20ins)	24.4%	Incomplete Testing	Chemo + IO
60	F	White	1	IVB	Current	LN	Point	*KRAS* G12C	0.2%	Incomplete Testing	Chemo + IO
61	F	Black	0	IVA	Current	LN	Point	*KRAS* G12C	2.9%	Incomplete Testing	Chemo + IO
62	F	White	1	IVA	Former	Lung	Point	*ERBB2* (Exon 7)	1.2%	Incomplete Testing	Chemo + IO
63	F	White	4	IVB	Former	Liver	Point	*KRAS* G12C	30.8%	Inadequate Panel	Hospice
79	F	White	1	IVA	Former	Bone	Insertion	*ERBB2* (Exon 20ins)	2.1%	Inadequate Panel	IO
78	F	White	3	IVB	Never	Lung	Point	*BRAF* V600E	1.0%	Inadequate Panel	Hospice
44	M	Asian	0	IVB	Former	Lung	Deletion	*EGFR* (Exon 18del)	2.2%	Inadequate Panel	Targeted

## 4 Discussion

The identification of AGAs is an essential component of optimizing outcomes for patients with mNSCLC, as the substantial therapeutic benefit of targeted agents can only be realized if AGAs are detected and acted on. While an emerging body of data and guidelines support concurrent tissue and plasma-based molecular sequencing, this practice has not been universally adopted and the mechanism underlying occasionally discordant tissue- and plasma-based sequencing results has not been well characterized. This study demonstrates that among patients with mNSCLC who have undergone comprehensive tissue-based sequencing, there remains a small but meaningful number of individuals for whom AGAs are detected only in plasma-based sequencing (10.4% in our sample), consistent with previously reported incremental detection rates of concurrent tissue- and plasma-based testing approaches, ranging from 5.2 to 15.3% [6,10,13].

In 72% (13/18) of cases in our cohort of non-squamous mNSCLC patients with AGAs detected exclusively in plasma-based testing, the discrepancy was due to inadequate solid tumor samples or orders for non-comprehensive sequencing panels. These data highlight that when obtaining sequencing in patients with mNSCLC, physicians must be cognizant of the type of assay being ordered, the class of alterations it is meant to detect, potential gaps in assessment, and confirmation that all requisitioned assays including DNA, RNA, and FISH testing are completed. However, the presence of “true tissue-negative” patients representing 28% (5/18) of our cohort with appropriate solid tumor testing completed on adequate samples, suggests that in select cases, the benefits of concurrent plasma-based testing extend beyond compensating for inadequate tissue samples. Plasma-based sequencing may aid in the detection of AGAs not captured by single-site biopsies potentially reflecting tumor heterogeneity in patients with bulky or metastatic disease, which may allow for more faithful representation of the genetics of a patient's malignancy. Ultimately, this comprehensive, concurrent tissue- and plasma-based testing approach, led to the appropriate utilization of efficacious targeted therapy in 3.5% (six patients) of our cohort of individuals with AGAs who underwent tissue- and plasma-based molecular testing. The established specificity of plasma-based sequencing in detecting AGAs and robust clinical responses to targeted therapy in this admittedly small subset of patients suggests these AGAs were true driver alterations^.^[[11], [12]] While plasma-based testing alone is unlikely to supplant tissue-based sequencing, it has a complementary role in mNSCLC patient evaluation that will continue to grow as assays improve.

Potential barriers to the wider utilization of concurrent sequencing approaches include cost, access, and provider familiarity with available plasma-based sequencing tests and comfort with result interpretation. The cost and accessibility of plasma-based sequencing approaches in particular, remain an issue that limits the global applicability of universal testing for mNSCLC patients, but we anticipate declining costs over time, and this approach has been shown to be cost effective in some healthcare systems, including the Canadian healthcare system [6]. A recent comparison of plasma-based sequencing approaches in advanced NSCLC in Singapore studied several approaches including tissue-based only, concurrent, and sequential testing [13]. This study found that currently, a sequential tissue-first based testing approach is the most cost-effective sequencing approach in their healthcare system. This finding is sensitive to targeted therapy and plasma-based NGS assay costs. As these costs decline, concurrent approaches may become more favorable in a wider range of economic settings [13]. Additionally, in this sample, concurrent testing captured the largest proportion of targetable alterations (94.2% vs. 92.3% with sequential testing) and may have particular utility in patients with technically challenging to biopsy or osseous-only metastases who are more likely to require repeat biopsies, leading to increased risk and cost [11,13].

There were no clear shared characteristics among individuals with AGAs detected exclusively in plasma-based sequencing in terms of patient demographics, mechanism of genetic alteration, or gene involved. Future research with a larger cohort may allow for a deeper understanding of the mechanisms that contribute to AGA detection in plasma-based sequencing alone and conversely the failure of molecular testing on tissue, which may facilitate a narrower application of concurrent molecular sequencing approaches. Our data, which demonstrate that this population is a molecularly and demographically heterogenous group, reinforces current guidelines supporting up-front concurrent tissue- and plasma-based molecular sequencing for all patients with mNSCLC with non-squamous histology.

## CRediT statement

Benjamin A Bleiberg: Conceptualization, Data curation, Formal analysis, Investigation, Methodology, Project administration, Writing – original draft preparation, Writing – review & editing.

Dylan G Scholes: Conceptualization, Data Curation, Formal analysis, Investigation, Writing – Original draft preparation, Writing – review & editing.

Lauren Reed-Guy: Methodology, Visualization, Writing – review & editing.

Devaki Dravid: Data curation, Investigation, Writing – review & editing.

Lova L Sun: Conceptualization, Supervision, Writing – review & editing.

Aditi P Singh: Conceptualization, Supervision, Writing – review & editing.

Roger B Cohen: Methodology, Supervision, Writing – review & editing.

Corey J Langer: Conceptualization, Supervision, Writing – review & editing.

Melina E Marmarelis: Conceptualization, Supervision, Writing – review & editing.

Charu Aggarwal: Conceptualization, Data curation, Methodology, Project administration, Supervision, Writing – review & editing.

## Ethical approval and patient consent document

This protocol was submitted for institutional IRB review, and it was deemed to meet eligibility for exempted status as a non-interventional, retrospective electronic-health record study. The IRB approval was granted to protocol #843355. Patient consent and waiver of HIPAA authorization requirements were not necessary due to the research protocol's exempted status. We instituted protocols to protect sensitive and identifying patient information per institutional protocols.
